# Fiber-Like Organization as a Basic Principle for Euchromatin Higher-Order Structure

**DOI:** 10.3389/fcell.2021.784440

**Published:** 2022-01-31

**Authors:** Amir N. Zakirov, Sophie Sosnovskaya, Ekaterina D. Ryumina, Ekaterina Kharybina, Olga S. Strelkova, Oxana A. Zhironkina, Sergei A. Golyshev, Andrey Moiseenko, Igor I. Kireev

**Affiliations:** ^1^ Department of Electron Microscopy, AN. Belozersky Institute of Physico-Chemical Biology, Lomonosov Moscow State University, Moscow, Russia; ^2^ Chair of Cell Biology and Histology, Biology Faculty, Lomonosov Moscow State University, Moscow, Russia; ^3^ Faculty of Bioengineering and Bioinformatics, Lomonosov Moscow State University, Moscow, Russia; ^4^ Laboratory of Electron Microscopy, Biology Faculty, Lomonosov Moscow State University, Moscow, Russia

**Keywords:** higher-order chromatin folding, euchromatin, replication, transcription, electron tomography

## Abstract

A detailed understanding of the principles of the structural organization of genetic material is of great importance for elucidating the mechanisms of differential regulation of genes in development. Modern ideas about the spatial organization of the genome are based on a microscopic analysis of chromatin structure and molecular data on DNA–DNA contact analysis using Chromatin conformation capture (3C) technology, ranging from the “polymer melt” model to a hierarchical folding concept. Heterogeneity of chromatin structure depending on its functional state and cell cycle progression brings another layer of complexity to the interpretation of structural data and requires selective labeling of various transcriptional states under nondestructive conditions. Here, we use a modified approach for replication timing-based metabolic labeling of transcriptionally active chromatin for ultrastructural analysis. The method allows pre-embedding labeling of optimally structurally preserved chromatin, thus making it compatible with various 3D-TEM techniques including electron tomography. By using variable pulse duration, we demonstrate that euchromatic genomic regions adopt a fiber-like higher-order structure of about 200 nm in diameter (chromonema), thus providing support for a hierarchical folding model of chromatin organization as well as the idea of transcription and replication occurring on a highly structured chromatin template.

## Introduction

In the interphase nucleus of higher eukaryotes, DNA displays up to 1,000-fold linear compaction by forming a complex with histones and a set of non-histone proteins—a chromatin. Despite many efforts aiming at elucidation of the DNA folding path in the nucleus, our understanding of how DNA is packaged into chromatin and adopts its conformation is still incomplete. The source of contradiction is rooted from the diversity of experimental techniques used to study chromatin organization at high spatial resolution. Initial attempts were made by analyzing permeabilized cells in order to improve chromatin contrast by removing soluble non-chromatin nuclear material (Zatsepina et al., 1983; Belmont et al., 1989). These studies revealed organization of chromatin into higher-order fibers of 100–130 nm in diameter—chromonema both in interphase and mitotic chromosomes. Careful ultrastructural analysis of cells entering and exiting mitosis demonstrated that mitotic chromosome compaction/decompaction is achieved by sequential folding/unfolding of chromonema into the fibers of even higher thickness supporting the idea of hierarchical DNA compaction (Belmont and Bruce 1994; Kireeva et al., 2004). These data were criticized on the grounds of possible artifactual chromatin aggregation caused by chromatin-compacting agents (divalent cations and/or polyamines). In contrast, various alternative approaches including live nucleosome tracking (Maeshima et al., 2014), ChromEMT (Ou et al., 2017), and cryo-electron microscopy (Eltsov et al., 2008, 2018; Nishino et al., 2012) that better maintain native chromatin structure failed to demonstrate any signs of hierarchical folding motifs beyond a nucleosome fiber. This has led to formulation of a “polymer melt” model of chromatin organization further supported by the concept of phase separation as a driving force of chromatin organization (Erdel and Rippe, 2018; Mirny et al., 2019). However, regardless of the exact way of DNA folding in the nucleus, it is generally accepted that the local ratio of DNA packaging tightly correlates with transcriptional activity, rendering chromatin subdivided into two fractions—transcriptionally active and centrally located euchromatin and permanently silent or transcriptionally repressed heterochromatin, which preferentially occupies perinucleolar and peripheral areas of the cell nucleus (Solovei et al., 2016; van Steensel and Belmont, 2017). Different chromatin fractions bear specific molecular signatures, such as characteristic histone post-translational modifications, sets of non-histone proteins, and patterns or DNA methylation, which cumulatively contribute to the maintenance of their structural and functional states.

To facilitate transcription, euchromatin is maintained in a more decondensed (“open”) state relative to heterochromatin as shown by both microscopy and biochemical assays, yet the degree of its “openness” apparently varies depending on the transcription level. On one extreme of the range lay highly transcribed chromosomal loci demonstrating complete chromatin unfolding, such as puffs in Diptera polytene chromosomes or loops in lampbrush chromosomes (Björk and Wieslander, 2015; Morgan, 2018). On the other hand, the majority of transcribed genes display much lower activity, raising the question whether euchromatin packaging displays variability depending on the transcription activity and what degree of compaction is most typical for it.

Direct imaging of euchromatic genomic loci at high resolution would make an ideal tool to answer this question. However, this approach faces several complications. First, identification of euchromatic loci merely by their relative positioning in the nucleus or by overall compaction state (Ou et al., 2017) may be misleading. Second, traditional ways of chromatin labeling [either by immunocytochemical approaches or by FISH (Boettiger et al., 2016)] require relatively mild fixation conditions required for probe penetration and antigene preservation, which are incompatible with maintenance of native chromatin structure, especially with FISH with its intrinsic harsh denaturation steps, while optimal fixation renders specific labeling ineffective and complicates the 3D analysis if applied to electron microscopy. Using an alternative approach based on analysis of transgenic loci labeled by the LacO/lac-repressor system and *in vivo* immunogold staining seems to preserve chromatin ultrastructure much better (Belmont et al., 2010). However, the artificial chromosomal loci may not faithfully recapitulate the behavior of endogenous genes upon transcription activation, while binding of tagged proteins to chromatin at high density required for good structural resolution may potentially disturb chromatin structure.

Previously, we adapted replicative labeling of DNA with 5-ethynyl-2′-deoxyuridine (EdU, Salic and Mitchison, 2008) to ultrastructural studies of chromatin reorganization during and after replication (Deng et al., 2016). Here, to specifically investigate structural organization of euchromatin with high spatial resolution and optimal structure preservation, we modified this approach based on prolonged replicative labeling to visualize long stretches of DNA (Visser and Aten, 1999) combining EdU labeling with biotin-streptavidin-mediated Nanogold detection scheme and electron tomography. We demonstrate here that the majority of early replicating euchromatin is arranged into higher-order fiber-like structures.

## Materials and Methods

### Cell Labeling and Fixation

HT1080 cells were plated on glass coverslips 1 day before the experiment. For labeling of replicated DNA, cells were incubated in 10 μM EdU (Thermo Fisher Scientific) for 2 h. Cells were fixed with prewarmed (37°C) 2.5% glutaraldehyde on cacodylate buffer (pH 7,2) for 1 h. After washing in PBS with 5 mM MgCl_2_ (PBS∗) three times for 5 min, free aldehyde groups were quenched with 20 mM glycine in PBS∗ (2 × 10 min). Cells were next permeabilized in 0.1% Triton X-100 in PBS∗ (PBS∗T) twice for 20 min and blocked in 1% BSA in PBS∗ for 1 h.

### EdU Detection

EdU was detected according to the Click-IT EdU Imaging Kit protocol with AlexaFluor488-azide or biotin-azide for 40 min. After EdU detection, cells were washed again with BSA-PBS*T buffer 3 times for 5 min, and then washed with deionized water 3 times for 5 min. Streptavidin-Nanogold (Nanoprobes) in BSA-PBS* T buffer (1:500) was then added to biotin-azide samples overnight and thoroughly washed with BSA-PBS*T buffer. For cells stained with AlexaFluor488-azide, processing for TEM included detection of AlexaFluor488 with mouse monoclonal antibodies against AlexaFluor488 (Thermo-Fisher) and Nanogold-conjugated goat anti-mouse Fab-fragments (Nanoprobes) at 1:400 dilution for 24 h with the same washes as for streptavidin-Nanogold staining. Cells were post-fixed with 1% glutaraldehyde in PBS buffer for 30 min, washed with 20 mM glycine in PBS* and deionized water and then free aldehyde groups were additionally quenched with NaBH_4_ (1 mg/ml) for 20 min and cells were extensively washed with several changes of deionized water. Fluorescently labeled samples were imaged with Eclipse Ti-E inverted microscope (Nikon) using 60 x 1.4 NA objective and appropriate filter sets. Z-stacks were recorded with Neo sCMOS camera (Andor) and deconvolved using NIS-Elements 5.3 software package.

### Ag-Amplification

Nanogold particles (1.4 nm) were silver-enhanced as described previously (Hainfeld and Furuya, 1992; Kireev et al., 2008). This procedure results in deposition of silver on the surface on Nanogold particles and formation of larger (10–20 nm) silver particles with an Au core, which are readily detected at low-magnification TEM. Briefly, 5 ml of 30% acacia powder solution in deionized water was mixed with 2 ml of 1 M MES (pH = 6.1) in a foil-wrapped 50-ml tube and mixed thoroughly for 30 min by slowly rocking the tube. Right before the procedure, 1.5 ml of freshly prepared 0.2% N-propyl gallate (Fisher) in deionized water was added to acacia powder mix and tube rocked for about 3 min, then 1.5 ml of freshly prepared 0.7% silver lactate in deionized water was added and a mix was rocked for another 2 min. In a dark room, the reaction mix was applied to the cells for 3–4 min and immediately washed out with several changes of deionized water.

### Dehydration, Epon Embedding, and Sectioning

Samples were dehydrated in a series of graded ethanol solutions and embedded in Epon 812 (Sigma-Aldrich). Coverslips were then removed by repetitive placement of the samples in liquid N_2_ and boiling water. The cells in early S-phase (replication pattern 1 or 2) were located under the bright-field microscope with 20 x lens and the blocks were manually trimmed with the razor blade. Sections that are 250–350 nm thick were cut on the Ultracut E ultramicrotome (Leica) in that serial sections were picked onto Formvar-coated 1-mm single-slot grids. Grids were either stained with 5% aqueous uranyl acetate for 20 min or left unstained, and then carbon-coated.

### Electron Tomography

Electron tomography data were acquired with a JEOL JEM-2100 200kV LaB6 transmission electron microscope, equipped with a Gatan GIF Quantum ER energy filter and SerialEM software (Mastronarde, 2005). Images were recorded in EFTEM mode with 20 eV energy-selecting slit, positioned at zero loss, near-parallel condenser illumination conditions, and 0.8 µm defocus. The tilt series was one-axis and one-directional from −60 to +60° with 2° steps. No dose-limiting procedures were carried out, but the tomography region was pre-illuminated with high electron dose. To estimate the resolution, we generated the pair of tomograms from even and odd tilt series subsets independently. The worst resolution estimate is 9 nm at 0.143 FSC criterion. The FSC was calculated across all tomograms including low SNR areas that do not contain nanoparticles.

### Tomogram Analysis

The tomogram reconstruction was performed with IMOD software (Kremer et al., 1996) following the standard workflow. Some Ag particles were selected as fiducials for accurate image alignment. All the subsequent steps were performed in ImageJ. During the first step, the default threshold was applied to the reconstructed tomogram to convert grayscale 3D-stack into a binary image. Then, the “3D Simple segmentation” plugin of the binary image was applied (with default threshold) to a binary image to identify 3D particles as objects.

Subsequent clusterization of those objects is necessary for further analysis of chromatin structure. First, local density was calculated with the “3D Density” plugin. This plugin requires two parameters: “number of neighbors” determines the number of closest neighbors to compute for each pixel, and “radius” determines the radius of expansion from the particle center (hence the radius of resulting fiber). 3D density was calculated for different radii with the number of neighbors set to 40 (variation of both parameters lead to somewhat similar results, so we chose to fix the number of neighbors and iteratively adjust the radius). The number of individual clusters was then plotted against the density calculation radius. The plateau on this plot indicates drastic change in density of golden particles, which can be interpreted as an edge of the object. Usually, two obvious thresholds could be clearly seen. 3D density maps calculated with those threshold radii were further converted to binary images using default brightness threshold. The resulting objects were considered as required chromatin structures.

In order to obtain some metrics of those fibers, first the “3D Distance map” plugin was used. It calculates minimum distance to the edge of the objects for every pixel and assigns it as brightness of this pixel. Since we were interested in overall fiber diameter, we needed to extract central pixels—axis—of the objects. This was achieved by the “Skeletonize 3D” plugin. Then, the skeletonized image and 3D distance map were combined, so that the final image contains only central axial pixels with their intensities indicating the radius (distance from the center to the edge of the object). Density histograms of these images show the distribution of fiber radii.

## Results

An important feature discriminating eu- and heterochromatin is replication timing. Microscopic analysis of the spatial distribution of replication foci during S-phase identified a set of specific patterns. These patterns appear in a rather strict temporal order during the genome replication with early patterns colocalizing with euchromatin while late patterns are similar to the distribution of heterochromatin (Nakamura et al., 1986; Manders et al., 1996; Zink et al., 1999). Tight correlation of the replication timing and replication patterns with transcriptional state was further confirmed by molecular approaches (Pope et al., 2013; Zhao et al., 2020). It was also shown that replicative domains (RDs) overlap with topologically associating domains (TADs) identified by Hi-C approach and representing DNA packaging units. The neighboring RDs replicate sequentially so that replication waves started at multiple points along the chromosome at the onset of the S-phase spread bidirectionally involving first euchromatic regions and then heterochromatin (Pope et al., 2014; Dileep et al., 2015).

In our previous works, we adopted EdU labeling protocol to electron microscopic detection of replicated DNA by using anti-AlexaFluor488 antibodies (Zhironkina et al., 2015; Deng et al., 2016). Using this approach, we obtained high-density labeling of replication foci after permeabilization. We used high-Mg buffer for stabilization of higher-order chromatin structure (Belmont et al., 1989), which allowed us to visualize chromonema fibers with high contrast and demonstrate that replication occurs on highly condensed chromatin template (Deng et al., 2016).

We also noted that early S-phase replication foci at the ultrastructural level represented densely labeled segments of chromonema fiber, suggesting that transcriptionally active chromatin might also fold into higher-order structures. However, since pre-fixation permeabilization of cells in chromatin-condensing conditions may possibly induce chromatin hyper-condensation or even artifactual aggregation, we decided to further investigate this question by developing an experimental approach for ultrastructural replicative labeling under conditions that maximally preserve native chromatin structure.

**FIGURE 1:**
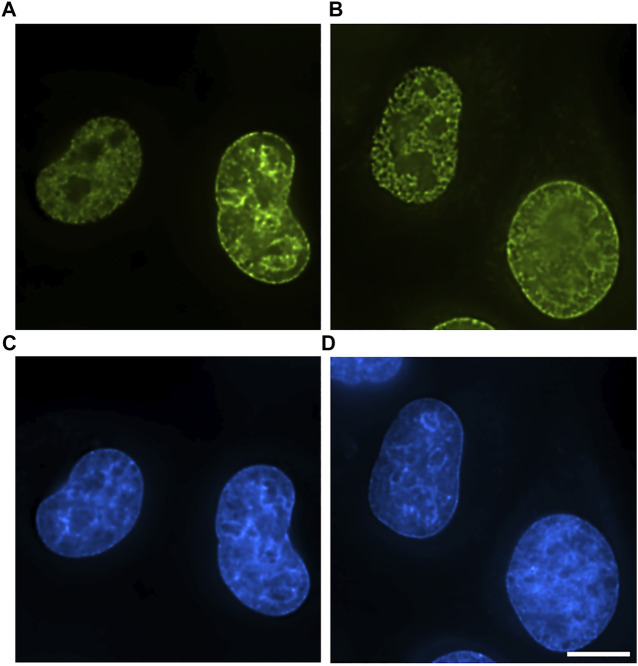
Comparison of labeling efficiency and probe penetration into EdU-labeled cells. **(A)** 10 min formaldehyde fixation, detection with AlexaFluor-azide. **(B)** 1 h glutaraldehyde fixation, detection with biotin-azide–streptavidin-AlexaFluor. Bar, 10 μm. **(C,D)** DAPI staining. Bar 10 μm.

First, we explored the possibility of application of antibody-mediated EdU labeling on cells fixed directly with gluteraldehyde. We found that gluteraldehyde fixation is widely used for electron microscopy of cells and tissues and superbly preserving cellular ultrastructure does not interfere significantly with Click-reaction if special care is taken to quench free aldehyde groups (Figure 1B). However, direct application of anti-fluorochrome antibodies demonstrated that glutaraldehyde fixation creates a diffusional barrier to antibodies as we observed clear gradient of labeling efficiency with peripheral chromatin labeled rather densely but the labeling density rapidly fading towards the nuclear interior (Supplementary Figure 2). To avoid this problem, we substituted fluorochrome-azide with biotin-azide and used Streptavidin-Nanogold for labeling. Reduction of the probe size and introduction of one-step labeling procedure allowed to obtain a uniform labeling throughout the nucleus while maintaining labeling intensity roughly at the same level with substantial gain in S/N ratio even in glutaraldehyde-crosslinked cells (Figures 1A,B).

**FIGURE 2:**
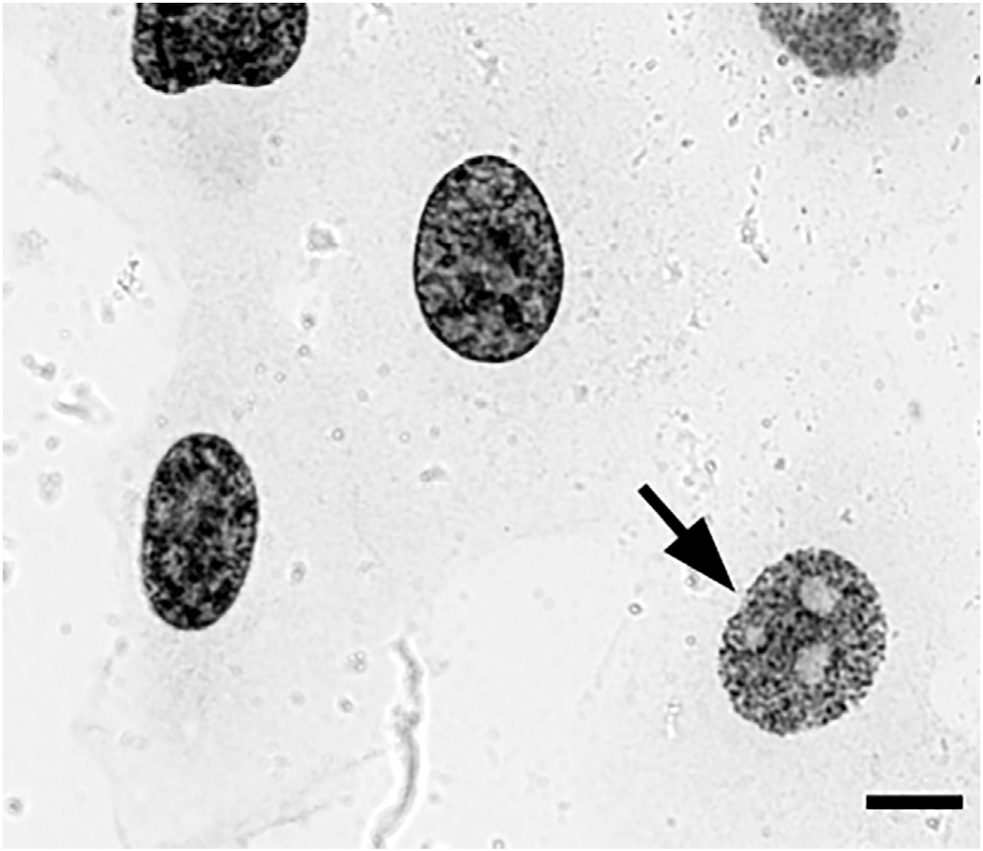
EdU pulse-labeled HT1080 cells after biotin-azide streptavidin-Nanogold detection and embedding demonstrate clear replication patterns en bloc under a transmitted light microscope, enabling selection of cells with early-S patterns (arrow) for sectioning. Bar, 10 μm.

**FIGURE 3:**
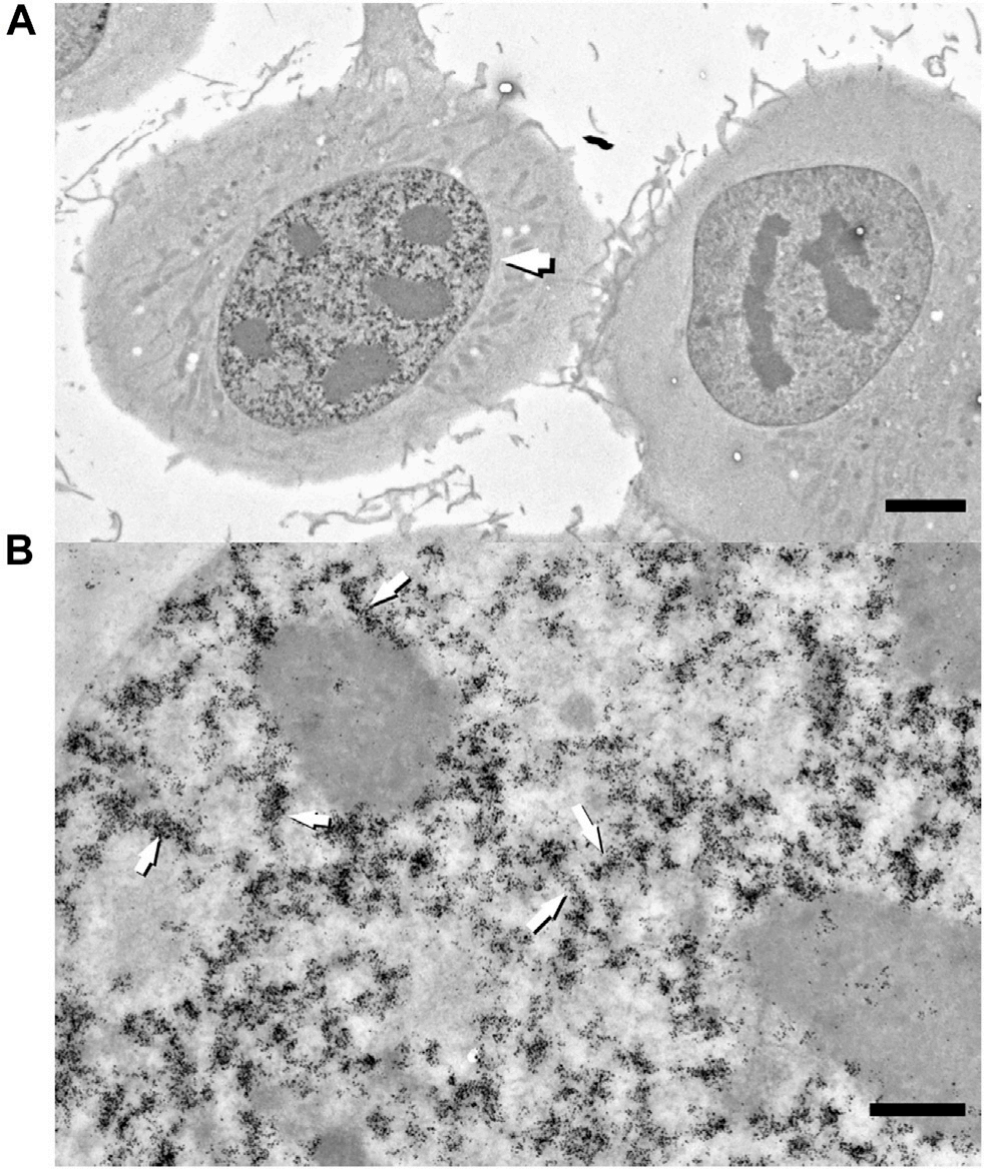
Raw zero-tilt image of a 250-nm-thick section of HT1080 cell in early S-phase labeled with 2 h pulse of EdU with subsequent detection with biotin-azide–streptavidin-Nanogold **(A)**. Segments of labeled fiber-like chromatin structures (arrows) are randomly distributed throughout nuclear interior. Bar, 1 μm **(A)** and 0.5 μm **(B)**.

**FIGURE 4:**
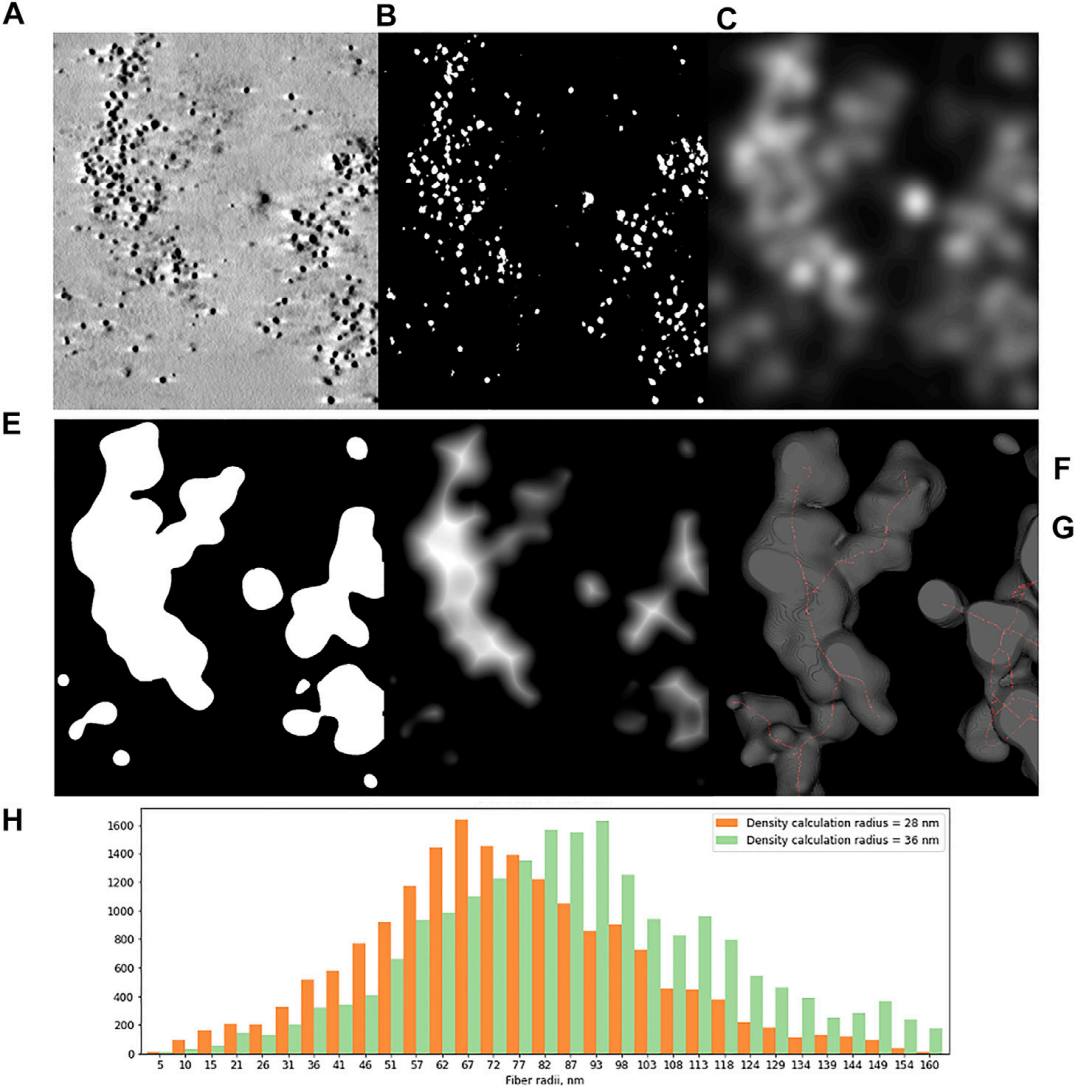
Tomographic slice of HT1080 cell nucleus labeled with 2 h pulse of EdU and detected with biotin-azide and streptavidin-Nanogold **(A)** and main steps of image analysis. **(B–F) (B)** Segmentation of Ag-enhanced Au particles. **(C)** 3D density calculation for different radii. The plot demonstrates total number of clusters depending on density calculation radius, green lines indicating the thresholds used for further calculations. **(D)** 3D density map. **(E)** Thresholded 3D density map. **(F)** 3D Distance map; Bar, 500 nm. **(G)** Histograms of local thickness distribution, calculated for two clustering thresholds show modal radii of higher-order chromatin fibers between 75 and 90 nm.

Next, we decided to test the hypothesis whether early-replicated euchromatin is organized into higher-order fibrillar structures. Since direct fixation does not allow to easily identify loosely packed chromatin on the background of non-chromatin nuclear components, we relied upon prolonged replicative labeling in order to visualize long DNA stretches representing several neighboring RDs and trace its folding based exclusively on amplified Nanogold particle distribution. For this reason, we chose 2-h EdU pulses, which would give a continuous labeling of at least three neighboring RDs [provided an average replication timing of a single domain in early-S estimated between 45 and 60 min (Ma et al., 1998)] while staying within a time frame of euchromatin replication that occurs during the first 3 h of S-phase in HT1080 cells (Deng et al., 2016). Since the estimates of a chromonema fiber (and RDs) size fall in the range between 100 and 200 nm (Zatsepina et al., 1983; Belmont et al., 1989; Belmont and Bruce 1994; Kireeva et al., 2004; Kireev et al., 2008; Rego et al., 2008; Cseresnyes et al., 2009; Baddeley et al., 2010; Su et al., 2020), in order to accurately measure the size of higher-order chromatin structures in 3D, we employed electron tomography of 300-nm plastic sections. The cells in early S-phase were selected for sectioning based on the pattern of RD distribution (Supplementary Figures S1A–E). The corresponding patterns are easily detected by bright-field microscopy after Ag amplification (Figure 2) and angular projections were collected from equatorial sections of the nuclei. Already on raw images, distribution of replicative label over the higher-order fibrillar chromatin structures becomes clearly seen (Figures 3A,B). The fibers were almost uniformly distributed over the nuclear interior excluding the nucleolus, as expected for euchromatin. Provided high labeling density, we could measure the fiber thickness by first calculating the silver-enhanced Au nanoparticle 3D density map on tomographic reconstructions using the ImageJ 3D Density plugin (Figure 4). The radius for counting the neighboring particles was determined by iteratively calculating 3D density map and measuring the number of objects after segmentation of the resulting map using the default threshold. A plateau on plots of the number of objects as a function of the radius chosen indicates a drastic change in density of golden particles (Supplementary Figure S3), which can be interpreted as an edge of the object. These radii were subsequently used for further calculations. In the majority of samples collected from five tomograms, two plateaus were detected (Supplementary Figure S4), indicating the existence of either two size classes or, most probably, variability in thickness within a single fibrillar structure.

Next, local thickness of the 3D objects representing segments of labeled chromatin higher-order structures was calculated by applying 3D Distance map and Skeletonize2D/3D plugins (Figure 4). The resulting histograms demonstrate the modal radius of the labeled fibers, calculated separately for two neighborhood radii, to be 74,5 nm (S.D. 26,77 nm, SE 0.2 nm) and 90,02 nm (S.D. 28,71 nm, SE 0.2 nm), which correspond to ∼150–180 nm fiber diameter.

## Discussion

In our previous work aimed at the analysis of transcription-dependent chromatin rearrangement using engineered chromosome loci containing inducible genes, we observed expected unfolding of BAC arrays upon transcription induction (Hu et al., 2009). However, estimations of linear compaction ratios based on measurements of FISH signals and direct immunoelectron visualization of the transgenes suggested that overall packing ratio (400- to 1,000-fold) of the transcribed loci remains well above expected for nucleosome fiber. This suggests that transcription may occur on highly condensed chromatin template. Obvious technical limitations of the approaches used and a desire to extend our study to endogenous loci have inspired us to design a method that would (1) allow for selective labeling of total euchromatin, (2) minimally perturb native chromatin structure, and (3) be easily compatible with high-resolution 3D analysis.

We decided to make use of metabolic DNA labeling with alkyne derivatives of nucleotides (EdU; Salic and Mitchison, 2008). When applied in the early S-phase, EdU is incorporated into transcriptionally active DNA, thus labeling euchromatin fraction. Since EdU detection with Click-chemistry does not require any DNA-perturbing treatments (denaturation enzymatic digestion, etc.), it is more compatible with high-resolution imaging compared to previously used halogenated nucleotides (Visser et al., 2000; Jaunin and Fakan, 2002). In our first attempt, we used a rather mild fixation protocol on pre-extracted nuclei and found that replication label in euchromatin at sites of replication and well after the replication is completed is distributed over distinct higher-order fiber-like structures (Deng et al., 2016). However, suboptimal fixation conditions used may potentially generate artifacts (Amiad-Pavlov et al., 2021) despite the attempts to stabilize chromatin structure with Mg^++^, so we decided to switch to more robust fixation protocol using glutaraldehyde in order to reproduce this label distribution in nearly intact chromatin. Generation of electron contrast for visualization of the label by TEM may be problematic since glutaraldehyde fixation, while optimally preserving nuclear ultrastructure (Fussner et al., 2012), limits large probe accessibility to the nuclear interior. Indeed, we mentioned dramatic differences in probe penetration between small fluorescently labeled azides and secondary gold-conjugated antibodies. DAB photooxidation as an alternative to gold particles (Ngo et al., 2016), although offering more uniform distribution in glutaraldehyde-fixed cells, gives much lower contrast and requires higher density labeling, which may disturb native chromatin organization. Thus, we switched to a multi-step protocol, which employs smaller-size components at each step, cumulatively achieving uniform labeling of chromatin throughout the nucleus. For this purpose, we used the biotin-streptavidin system to link EdU to Nanogold particles with subsequent silver enhancement for high contrast labeling detectable both at low-magnification optical microscopy and TEM. The ability to discern label distribution with bright-field optical microscopy greatly facilitated pre-selection of nuclei with labeled euchromatin for sectioning and tomography. This protocol has several advantages over previously published ones (Vogel et al., 1990; Jaunin and Fakan, 2002; Philimonenko et al., 2004; Koberna et al., 2005; Deng et al., 2016). Glutaraldehyde fixation ensures optimal preservation of chromatin near-native structure. Click-chemistry provides simple and extremely selective labeling of replicated DNA, without the need of DNA denaturation prerequisite for BrdU detection with antibodies used in previous reports. The use of streptavidin-Nanogold conjugates provides better penetration efficiency even into glutaraldehyde-fixed samples due to the relatively small size of the probe. Overall our protocol allows for high-contrast high-efficiency pre-embedding labeling compatible with various 3D-electron microscopy techniques.

This protocol has allowed us to visualize continuously labeled segments of the genome corresponding to euchromatin, in three dimensions. Although relatively large size of silver particles does not allow for tracing nucleosome chain folding paths as offered by ChromEMT (Ou et al., 2017) or cryoET (Eltsov et al., 2018), we achieved a satisfactory labeling density for visualization of higher-order chromatin structures more than 100 nm thick. Surprisingly, we found that the majority of chromatin labeled in early S-phase is forming fiber-like structures of the thickness ranging from 130 to 200 nm. These estimations closely correlate to the measurements obtained in the cells permeabilized in chromatin-stabilizing conditions (Belmont and Bruce 1994; Kireeva et al., 2004; Rego et al., 2008), as well as both *in vivo* and *in situ* labeled engineered chromosome loci (Strukov et al., 2003; Kireev et al., 2008; Hu et al., 2009), further supporting the idea of hierarchical folding principle of chromatin organization and suggesting that this multi-step folding is characteristic not only for highly condensed repressed heterochromatin, but also for transcriptionally active euchromatin. At the first glance, this idea contradicts a generally accepted principle of correlation between transcription activity and chromatin compaction. However, various experimental approaches have demonstrated that chromatin displays a high degree of local dynamics, which would allow for both accessibility of the genes for transcription-related *trans*-factors and local and temporal DNA conformational changes required for transcription and, more broadly, for any type of activity involving DNA (replication, repair, etc.) (Deng et al., 2016; Nozaki et al., 2018). We can propose this dynamics to occur within higher-order chromatin domains, which are aligned in the cell nucleus into chromonema fibers. One can argue that apparent fiber-like structures may result from an exclusion of euchromatic domains from chromosome territories, as proposed by the model of interchromatin domain (Cremer et al., 2020). However, high-resolution FISH analysis of contiguous DNA segment containing transcriptionally active genes rather suggests a fiber-like folding (Volpi et al., 2000; Muller et al., 2004; Goetze et al., 2007; Hu et al., 2009).

Chromatin dynamics experiments, as well as high-resolution tracing of nucleosome chains *in situ* and x-ray scattering, suggest the absence of any regular folding of 10-nm nucleosome fiber (Fussner et al., 2012; Nishino et al., 2012; Ricci et al., 2015; Nozaki et al., 2017; Ou et al., 2017). Yet, higher-order chromatin fiber-like structures may be formed by irregularly packed nucleosome chains, represented by a series of DNA loops of varying size, whose state of compaction is controlled by phase-separation mechanisms and/or a combination of loop-forming activities. Recent studies including Hi-C and single-molecule imaging suggested the dynamic nature of the loops with cohesin complex serving as both a loop-forming and a loop-maintaining factor (Gassler et al., 2017; Wutz et al., 2017; Nuebler et al., 2018; Davidson et al., 2019). A dynamic loop extrusion process further contributes to the variability in overall linear parameters of the chromonema fibers, partially explaining a rather wide range of thicknesses measured on electron tomograms. Fluctuations of local transcription activity may represent another source of variability in chromonema structure, which would require more accurate assessment of chromatin folding of individual genes at high resolution and maximal structural preservation.

## Data Availability

The raw data supporting the conclusion of this article will be made available by the authors, without undue reservation.
