# Supplementary figures and images for: Fiber-Like Organization as a Basic Principle for Euchromatin Higher-Order Structure
Source: Front Cell Dev Biol. 2022 Jan 31;9:784440. doi: 10.3389/fcell.2021.784440 (PMC8841976; doi:10.3389/fcell.2021.784440)

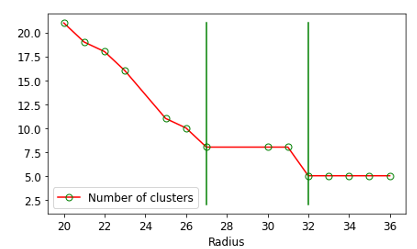

Supplement: Supplementary file 1 [file Image3.TIF]

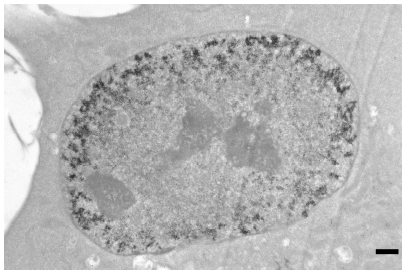

Supplement: Supplementary file 2 [file Image2.TIF]

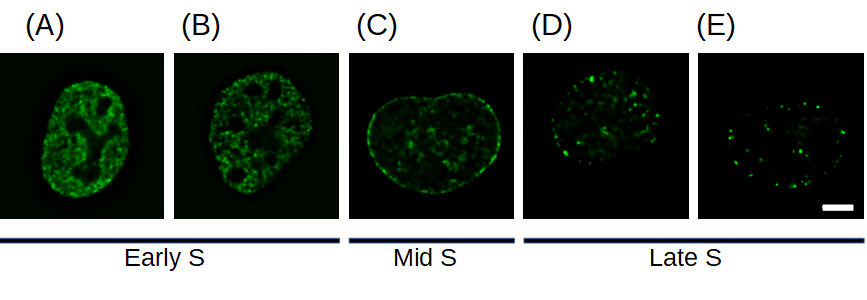

Supplement: Supplementary file 3 [file Image1.TIF]
